# *HNF1α* defect influences post-prandial lipid regulation

**DOI:** 10.1371/journal.pone.0177110

**Published:** 2017-05-11

**Authors:** Matthieu St-Jean, François Boudreau, André C. Carpentier, Marie-France Hivert

**Affiliations:** 1 Department of Medicine, University of Sherbrooke, Sherbrooke, Quebec, Canada; 2 Department of Anatomy and Cellular Biology, University of Sherbrooke, Sherbrooke, Quebec, Canada; 3 Department of Population Medicine Harvard Medical School, Harvard Pilgrim Health Care Institute, Boston, Massachusetts, United States of America; 4 Diabetes Unit, Massachusetts General Hospital, Boston, Massachusetts, United States of America; University of Barcelona, Faculty of Biology, SPAIN

## Abstract

**Purpose:**

Hepatocyte nuclear factor 1 alpha (*HNF1α*) defects cause Mature Onset Diabetes of the Young type 3 (MODY3), characterized by defects in beta-cell insulin secretion. However, *HNF1α* is involved in many other metabolic pathways with relevance for monogenic or polygenic type 2 diabetes. We aimed to investigate gut hormones, lipids, and insulin regulation in response to a meal test in *HNF1α* defect carriers (MODY3) compared to non-diabetic subjects (controls) and type 2 diabetes (T2D).

**Methods:**

We administered a standardized liquid meal to each participant. Over 6 hours, we measured post-meal responses of insulin regulation (blood glucose, c-peptide, insulin), gut hormones (ghrelin, glucose-dependent insulinotropic polypeptide, glucagon-like peptide-1) and lipids (non-esterified fatty acids [NEFA] and triglycerides).

**Results:**

We found that MODY3 participants had lower insulin secretion indices than controls and T2D participants, showing the expected β-cell defect. MODY3 had similar glycated hemoglobin levels (HbA1c median [IQR]: 6.5 [5.6–7.6]%) compared to T2D (median: 6.6 [6.2–6.9]%; *P*<0.05). MODY3 had greater insulin sensitivity (Matsuda index: 71.9 [29.6; 125.5]) than T2D (3.2 [4.0; 6.0]; *P*<0.05). MODY3 experienced a larger decrease in the ratio of NEFA to insulin (NEFA 30–0 / insulin 30–0: -39 [-78; -30] x10^4^) in the early post-prandial period (0–30 minutes) compared to controls and to T2D (-2.0 [-0.6; -6.4] x10^4^; *P*<0.05). MODY3 had lower fasting (0.66 [0.46; 1.2] mM) and post-meal triglycerides levels compared to T2D (fasting: 2.3 [1.7; 2.7] mM; *P*<0.05). We did not detect significant post-meal differences in ghrelin and incretins between MODY3 and other groups.

**Conclusion:**

In response to a standard meal test, MODY3 showed greater early post-prandial NEFA diminution in response to relatively low early insulin secretion, and they maintained very low post-prandial triglycerides levels.

## Introduction

Hepatocyte nuclear factor 1 alpha (HNF1α) is expressed in the epithelium of multiple organs such as kidney, pancreas, intestine and liver and acts as a transcription factor for the expression of several genes such as hepatic albumin, α-1 antitrypsine, β-fibrinogen, and C-reactive protein[1],[2]. Mice that are homozygote for *HNF1α* deletion present a particular phenotype characterized by hepatic enlargement, phenylketonuria, Fanconi syndrome and non insulin-dependent diabetes[3]. *HNF1α* functional mutations in human are associated with monogenic diabetes known as Mature Onset Diabetes of the Young type 3 (MODY3); this condition follows an autosomal dominant transmission (affected individuals carrying only one defective allele) and has a milder phenotype than homozygote knockout mice models. Common variants in *HNF1α* are also associated with increased risk of developing type 2 diabetes (T2D) [4]. One of the major manifestations of *HNF1α* defect in humans is β-cell dysfunction that impairs glucose-induced insulin secretion[5],[6]. *HNF1α* defect in humans is also associated with increased bile acid synthesis[7], and influences levels of total cholesterol and low-density lipoprotein[8]. Studying carriers of *HNF1α* defects could therefore contribute to a better understanding of diabetes pathophysiology and related metabolic pathways, and open doors to develop new therapies for this disease that now affects about 12–14% of the USA population and is predicted to continue to increase in prevalence in the coming years[9].

In the last decade, many emerging anti-diabetic drugs were related to gut hormones because of their contribution to the pathophysiology of obesity and T2D[10]. The most studied of these hormones are glucose-dependent insulinotropic polypeptide (GIP) and glucagon-like peptide-1 (GLP-1), which are implicated in post-prandial glucose homeostasis[11]. Ghrelin, another gastrointestinal hormone, produced mainly in the stomach, intestine and pancreas is also studied for possible implication in diabetes[12]. Ghrelin also plays a role in glucose homeostasis[13]: it may induce hyperglycaemia and diminish insulin secretion by binding to the GHS-R on β-cells[14]. Conflicting results have been reported for the levels of acylated ghrelin in T2D[15]-[16]. In mouse models, ghrelin mRNA increased almost four-fold in homozygous *HNF1α* knockout mice compared to wild type and was associated with decreased insulin level[17]. Furthermore, the use of an antagonist of ghrelin, (D-Lys3)-GHRP-6, markedly lowered blood glucose by improving insulin secretion in diabetic *HNF1α* knockout mice[18]. Given this impressive glycemic response to a ghrelin antagonist in this *HNF1α* knockout mice model, we wanted to investigate the pathophysiology of ghrelin variations in MODY3 patients. Indeed, very little is known about the levels of gut hormones, including ghrelin and incretins, in humans affected by MODY3. Investigating fasting and post-prandial hormonal, glycemic, and lipids regulation might inform us on the pathophysiology of glucose regulation in both monogenic and polygenic diabetes.

The objective of this study was to investigate hormonal response to a standardized meal test in MODY3 compared to healthy subjects and T2D patients. Since we know that lipids contribute to diabetes pathophysiology and related complications and that MODY3 have a higher risk of diabetes macrovascular complications than the other types of MODY[19], we further evaluated post-prandial lipid profiles including non-esterified fatty acid (NEFA) and triglycerides in MODY3 patients.

## Subjects and methods

### Subjects

We recruited 3 groups of individuals: healthy subjects without diabetes (controls, n = 9, 4 male, 5 female), T2D patients (n = 8, 6 male, 2 female), and MODY3 with classic mutations (n = 5, 3 male, 2 female). The study was approved by the ethics committee for research on humans of the Research Center of the Centre Hospitalier Universitaire de Sherbrooke (CHUS). We obtained written informed consent from all subjects. Healthy men and non-pregnant women were invited to participate as part of the control group; they were excluded if they were taking medication that could affect glucose or lipid homeostasis. We recruited patients with T2D following referral by endocrinologists from CHUS. Inclusion criteria for T2D group were: a) confirmed diagnosis of T2D based on the American Diabetes Association guidelines[20], b) treated with diet, metformin, and/or sulfonylurea c) men and non-pregnant women. All T2D patients were taking metformin with a median dose of 1850 mg/day (IQR; [1000–2000]); 4 patients were additionally taking gliclazide with a median dose of 140 mg/day (IQR; [90–190]). We instructed patients to take only metformin on the morning of the meal test and to suspend other drugs until the end of the test; we made this decision because we wanted to avoid both overly hyperglycemic status at meal intake and modulation of the insulin response by sulfonylurea. Five T2D patients were also on statins and 8 were on anti-hypertensive drugs.

MODY3 were identified among patients with known mutations and confirmed genotype (C.872insC in 2 patients and P.arg159trp in 3 patients) in clinical electronic medical records of the CHUS. All cases identified were invited to participate through contact with their attending physician. Additional inclusion criteria were: a) age ≥ 5 years old b) men and non-pregnant women. We included 2 young HNF1*α* defect carriers who had not reach diabetes status to maximize our sample size given the low prevalence of this condition. For patients under insulin treatment (n = 2), instructions were provided to administer long acting insulin the night before test day (to avoid overly hyperglycemic status at the beginning of the meal test intake), but to refrain from taking any short acting insulin the morning of the meal test. By avoiding taking short acting insulin, we expect that the insulin response to the meal test is the intrinsic β-cell capacity to secrete insulin in our MODY3 participants.

### Methods–clinical and biochemical assessments

During the initial visit, we collected clinical information including socio-demographics, medical history and medications. On the same day, we measured vital signs: blood pressure, heart rate and oxygen saturation. If participants met inclusion criteria, we scheduled a second visit for the experimental meal test (1–3 weeks after initial visit). We instructed participants to fast for at least 10 hours before presenting on the experimental day.

During the experimental visit, we administered a standardized meal test (905 kcal: 33% of fat, 50% carbohydrates and 17% proteins) as a liquid shake; quantity (mL) was adjusted for individuals less than 18 years of age based on a calculation of their estimated energy need (X (mL) = (Estimated Energy Need (EEN)) X 1000/(2130 x 3) in which EEN (kcal) = 88.5 –(61.9 x age (years)) + [(26.7 x weight (kg)) + (903 x height (m))] + 20). We measured height with a stadiometer and weight at fasting with a Tanita body composition scale. An I.V. catheter was inserted for blood sampling. A baseline blood sample was drawn just before meal intake, and then every 30 minutes after meal intake over 6h during the post-meal period without any other food/liquid intake permitted. In all samples collected (pre and post-meal), we measured plasma glucose, insulin, C-peptide, NEFA, triglycerides, total GLP-1, total GIP and acylated ghrelin. In addition, we measured A1c on collected fasting samples.

Glucose levels were determined in real-time by enzymatic method with hexokinase (Cobas 6000, Roche, CV = 3%). A1c was measured using mass liquid chromatography (Tosoh 7, Tosoh Bioscience, CV < 2.6%). All other samples were stored at -80°C until dosage (performed within the same assays for all subjects). For total GIP and total GLP-1, blood was collected in EDTA tubes with Dipeptidyl Peptidase-IV (DPP-IV) inhibitor to avoid their breakdown. Insulin, C-peptide, total GLP-1 and GIP were measured by Luminex-ELISA (HMHEMAG-34K, Millipex Map, CV < 10%). Acylated ghrelin was measured with Radioimmunoassay (Linco’s Ghrelin (active) RIA kit, # cat: GHRA-88HK, within CV < 10%) from blood collected with aprotinine. Triglycerides and NEFA were measured with colorimetric methods (Infinit 200, Tecan, (Wako: HR series NEFA-HR and Wako: L-Type Triglycerides), CV < 10%).

### Post-meal excursions

We calculated post-meal excursion of each biomarker using the area-under-the-curve (AUC) formula: 15*(T_0_+2*(T_30_+…+T_330_)+T_360_). We imputed missing values using different strategies, depending on the time point at which the value was missing. We used a linear interpolation when the shape of the curve allowed us to estimate that the missing point would be about half way between two existing points. When we could not assume linearity for the missing point, we replaced it by the group mean (control, T2D, or HNF1*α*) of the specific biomarker at this time point. When missing values were at the end of the curve, we used two different techniques: if levels of a biomarker were stable at the end of the curve, we carried forward the last observation; when levels were going downward or upwards, we replaced the missing value by linear approximation.

### Calculations for insulin sensitivity and secretion indices:

Insulin sensitivity/resistance was determined by two different methods. First, the Matsuda index was used to establish insulin sensitivity from test meals using the validated formula[21]. We also calculated the homeostasis model for insulin resistance (HOMA-IR): [fasting insulin (mU/mL) x fasting blood glucose (mM)]/22,5[22].

We estimated insulin secretion using multiple validated indices. The insulinogenic index was calculated as the Δ insulin 30–0 min/ Δ glucose 30–0 min (pM/mM))[23]. The insulin secretion rate (ISR) was determined by deconvolution of plasma C-peptide levels with standard two-compartmental kinetic parameters as we previously published[24],[25]. The Insulin Secretion Index (ISI) was calculated as the ISR AUC/glucose AUC over the first 30 min of the post-meal period. The Disposition Index (DI), an integrated index of β-cell function in face of insulin sensitivity levels, was calculated as ISI x Matsuda index[26].

### Statistical analysis

Indices of insulin sensitivity or secretion, AUCs, and fasting biomarker values are expressed as median and interquartile range (IQR). We used the Mann-Whitney non-parametric test to evaluate differences between baseline characteristics (BMI, age and HbA1c), fasting biomarkers, insulin sensitivity/secretion indices and biomarkers’ AUCs. Dichotomous characteristics were compared using exact Fischer tests. We considered two-sided P values < 0.05 significant. Data were analyzed using IBM SPSS statistics 22 software (IBM, Armonk, NY, USA).

## Results

### Baseline characteristics (Table 1)

MODY3 participants were similar in age and gender ratio compared to controls but were younger than T2D. MODY3 participants had similar A1c to T2D and both groups had higher A1c than controls, as expected. MODY3 participants had a lower median BMI than controls or T2D participants, keeping in mind that two out of five of MODY3 subjects were younger than 18. Defining obesity as BMI>30kg/m^2^ in adults or BMI >95^e^ percentile in paediatric populations, we had 1 participant with obesity in the MODY3 group (1 out 5) and 1 participant with obesity in the control group (1 out 9).

**Table 1:** Baseline characteristics of participants.

Groups	Controls	MODY3	Type 2 diabetes (T2D)
N	9	5	8
	Median [IQR] or N (%)	Median [IQR] or N (%)	Median [IQR] or N (%)
Age (years)	25.0 [23.5–28.0]	27.0 [7.5–47.5]	54.0 [43.8–65.0]
Sex (M/F)	4/5	3/2	6/2
A1c (%)	5.1 [5.1–5.3]	6.5 [5.6–7.6]^1^	6.6 [6.2–6.9]
BMI (kg/m^2^)	25.2 [23.9–27.6]	19.1 [14.2–25.5]°^1^ ^2^	39.9 [31.2–47.9]
N (%) obesity in groups (BMI > 30 or >95th percentile BMI z-score)	1 (11.1)	1 (20.0)^2^	7 (87.5)

Continuous values are expressed as median and Interquartile Range (IQR)

° includes crude BMI of two individuals <18 years old

1: HNF1α different than controls (p < 0.05)

2: HNF1α different than T2D (p < 0.05)

### Glycaemia, insulin and C-peptide regulation indices (Table 2, Fig 1A, 1B and 1C)

**Fig 1:**
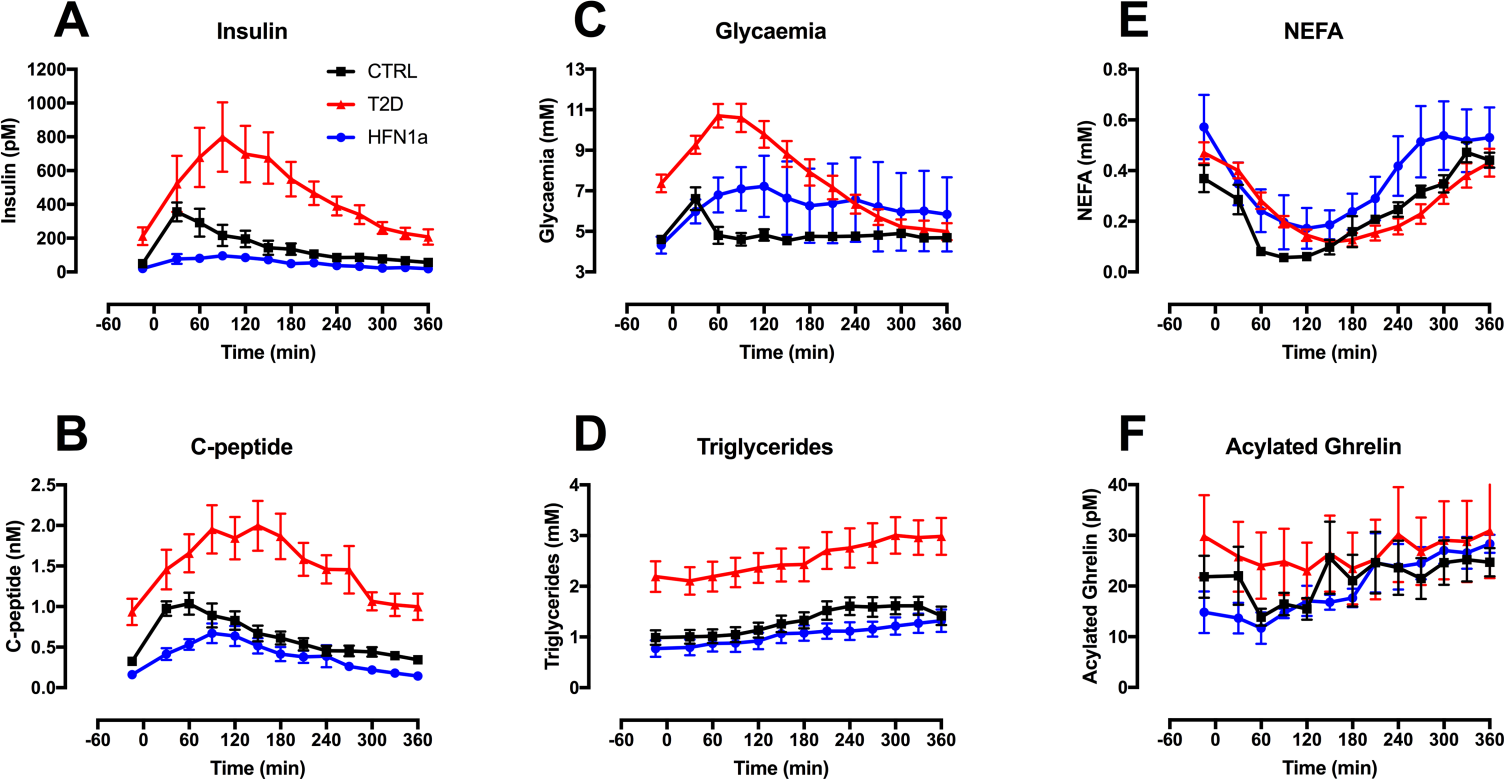
Post-meal excursions of biomarkers in HNF1α, control, and type 2 diabetes groups. (A) Insulin (B) C-peptide (C) Glycemia (D) Triglycerides (E) NEFA (F) Acylated Ghrelin.

We found no significant difference in fasting glucose between MODY3 and control groups, keeping in mind that the two children in the MODY3 group have not yet met the clinical definition of diabetes diagnosis. In the post-meal period, MODY3 post-prandial excursion of glucose (AUC glucose) was not statistically different from controls, once again keeping in mind the two children in the MODY3 group. However, the post-meal variation graphic (Fig 1C) illustrates a delay in decrease in post-meal glycaemia in MODY3 participants compared to controls. Fasting C-peptide was lower in the MODY3 group than controls. MODY3 participants showed lower fasting glucose, insulin and C-peptide levels than T2D (all *P*-values <0.05). Both the Matsuda index and HOMA-IR showed that MODY3 participants had much greater insulin sensitivity (or lower insulin resistance) than T2D (*P*-values <0.05).

**Table 2:** Differences in glycaemic, insulin regulation indices, lipids and incretins in fasting and during post-prandial between HNF1α and controls or type 2 diabetes.

	Controls	MODY3	Type 2 Diabetes (T2D)
**Glucose, insulin and C-peptide basal and post-prandial**			
Fasting glucose (mM)	4.4 [4.3; 5.1]	3.9 [3.6; 5.3] ^2^	7.6 [6.3; 8.5]
Fasting insulin (pM)	29.1 [26.1; 52.8]	17.8 [5.7; 37.8]^!^ ^2^	169.4 [129.9; 223]
Fasting C-peptide (nM)	0.31 [0.23; 0.36]	0.16 [0.11; 0.21] ^1^^,^^2^	0.89 [0.57; 1.3]
AUC-glycaemia (mM x 360 min)	1731.4 [1597.0; 1955.3]	1858.5 [1565.9; 3220.5]	2821.5 [2493.4; 3172.5]
AUC-Insulin (pM x 360 min)	41094.9 [28661.1; 75783.8]	15445.4 [10538.7; 29528.5] ^1^^,^^2^	138830.5 [118321.1; 251681.4]
AUC-C-peptide (nM x 360 min)	214.4 [144.0; 298.2]	149.5 [102.5; 180.2] ^2^	565.6 [463.0; 723.3]
**Insulin secretion indices**			
Δ glucose 30–0 min (mM)	1.8 [0.9; 2.3]	2.1 [0.4; 2.7]	1.9 [1.2; 2.9]
Δ insulin 30–0 min (pM)	277.6 [194.6; 422]	31.8 [16.8; 110.2]^1^^,^^2^	174.9 [121.5; 384.9]
Δ C-peptide 30–0 min (nM)	0.59 [0.50; 0.84]	0.14 [0.11; 0.46]^1^	0.49 [0.29; 0.71]
Insulinogenic index	180.7 [106.8; 372]	35.3 [15.4; 180.6]^1^	125.1 [49.2; 363.4]
Insulin Secretion Index	42.1 [38.2; 64.8]	15.0 [11.6; 19.6]^1^^,^^2^	44.7 [37.3; 85.2]
**Insulin resistance/sensitivity indices**			
HOMA-IR	0.86 [0.73; 1.37]	0.69 [0.26; 0.99] ^2^	8.6 [4.7; 11.3]
Matsuda index	30.1 [18.5; 36.4]	71.9[29.6; 125.5]^2^	3.2[4.0; 6.0]
**Disposition Index**			
DI (ISI*Matsuda)	1232 [848.3; 1654]	1127 [512.1; 1652]^2^	153.2 [137.8; 375.5]
**Fatty acid regulation**			
Fasting Triglycerides (mM)	1.0 [0.6; 1.1]	0.66 [0.46; 1.2] ^2^	2.3 [1.7; 2.7]
Fasting NEFA (mM)	0.33 [0.25; 0.55]	0.71 [0.28; 0.80]	0.49 [0.35; 0.56]
Δ NEFA 30–0 min (mM)	-0.08 [-0.13; -0.06]	-0.21 [-0.38; -0.09]	-0.06 [-0.15; -0.01]
Δ NEFA 30–0 min/ Δ insulin 30–0 min (*10^4^ (mM/pM))	-3.4 [-4.7; -2.0]	-39 [-78; -30]^1^^,^^2^	-2.0 [-0.6; -6.4]
Δ NEFA 30–0 min/ Δ C-peptide 30–0 min (mM/nM)	-0.13 [-0.24; -0.08]	-0.93 [-1.6; -0.53] ^1^^,^^2^	-0.19 [-0.43; -0.02]
AUC-NEFA (mM x 360 min)	80.2 [70.2; 83.7]^!^	126.2 [68.4; 184.9]	83.2 [68.3; 114.2]
AUC-TG (mM x 360 min)	500.1 [366.1; 552.4]	350.4 [280.5; 484.8] ^2^	971.6 [617.3; 1178.9]
**Ghrelin and incretins**			
Fasting Total GIP (pM)	8 [5; 9.6]	9 [7.9; 17.2]	17.6 [13.1; 21.7]
Fasting Total GLP-1 (pM)	20.4 [15.1; 47.3]	11 [7.1; 33.9] ^2^	55.6 [36.6; 89.2]
Fasting Acylated ghrelin (pM)	19.6 [13; 31.1]	15.1 [7.8; 21.7]	20.5 [13.9; 54.6]
AUC-Total-GIP (pM x 360 min)	20885.1 [19004.2; 26238.8]	27480.0 [13808.9; 29202.2]	19348.8 [16663.8; 22839.6]
AUC-Total-GLP-1 (pM x 360 min)	18476.6 [13506.3; 27090.2]	17446.5 [15624.1; 23572]	27219.8 [19533.7; 36174.8]
AUC-Acylated-Ghrelin (pM x 360 min)	6370.5 [4536.1; 10794]	6741.5 [5714.8; 8882.1]	6776 [3990.7; 16668]

Expressed as median and Interquartile Range (IQR)

! missing 1 value

1: HNF1α different than controls (p < 0,05)

2: HNF1α different than T2D (p < 0,05)

Formulas:

Insulinogenic index = Δ insulin 30–0 min/ Δ glucose 30–0 min

Insulin secretion index = ISR (C – peptide deconvolution 0–30 min)/AUC glucose (0–30 min)

HOMA-IR = Glucose (mM) x insulin (mu/L) / 22,5

Matsuda index: $\frac{10 000}{\sqrt{(\text{Gluc}0\text{minxinsulin}0\text{minx}(\text{mean Gluc}\;0\text{-}120\text{min})\text{x}(\text{mean insulin}\;0\text{-}120\text{min}))}}$

### Insulin secretion indices (Table 2)

In the early post-prandial state, we observed an absence of insulin and C-peptide peaks at 30 minutes in MODY3 participants when compared to controls (Fig 1A and 1B), showing the deficient early phase insulin secretion of these patients. The Insulinogenic Index (Δinsulin (30–0)/Δglucose (30–0)) was significantly lower in MODY3 (median [IQR]: 35.3 [15.4; 180.6]) than controls (180.7 [106.8; 372.0]; *P* = 0.039). The ISI, calculated with C-peptide deconvolution over the first 30 min of the post-meal period, also shown the diminished insulin secretion capacity of MODY3 β-cells (median [IQR]: 15.0 [11.6; 19.6]) compared to controls (42.1 [38.2; 64.8]; *P*<0.001) and T2D (44.7 [37.3; 85.2]; *P*<0.03). Other markers of post-prandial insulin secretion (insulin or C-peptide levels over first 30 min or AUC over 360 min) were also lower in the MODY3 group compared to the T2D or control groups (Table 2). These observations fit with the insulin secretion defect reported previously in literature.

**Fig 2:**
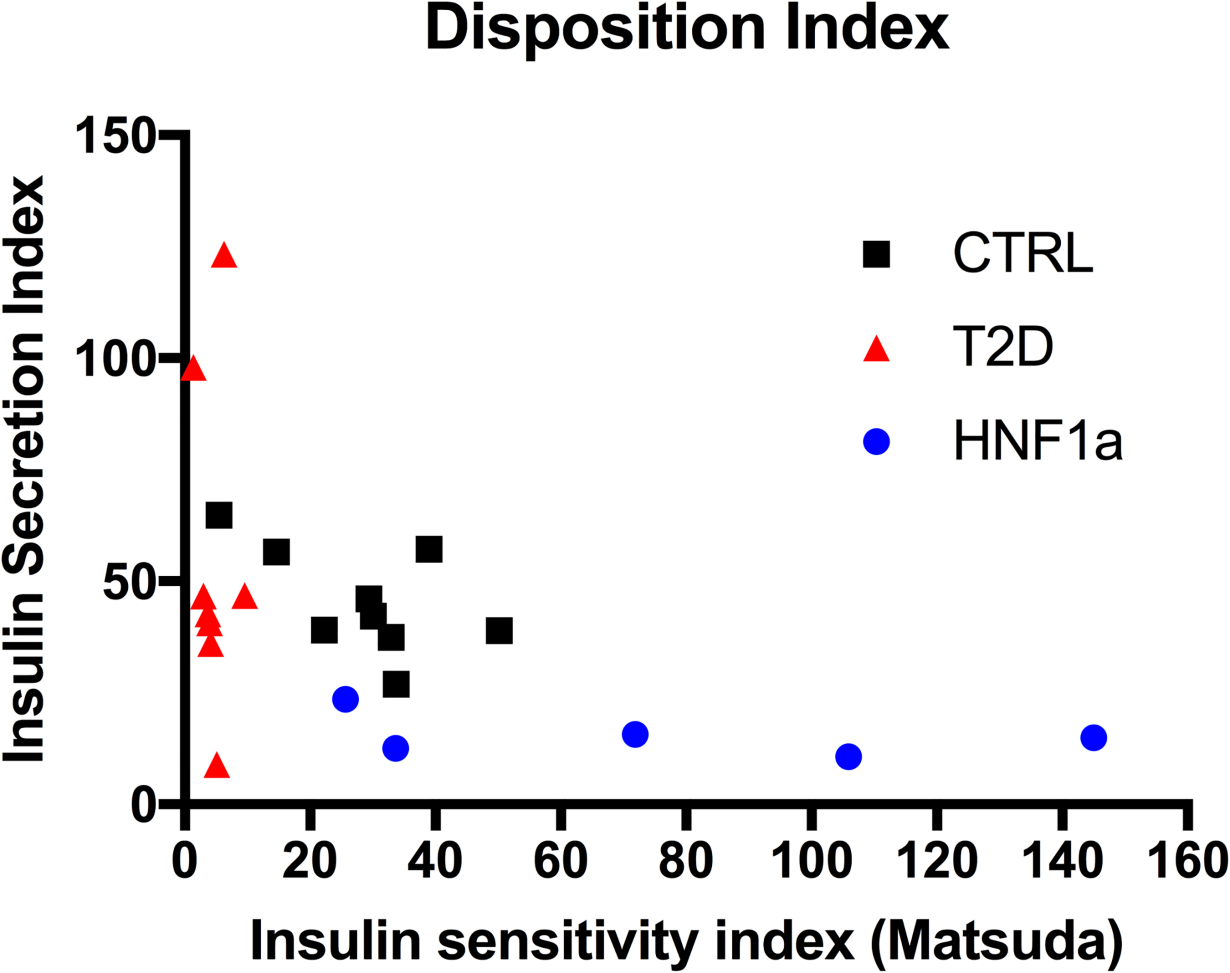
Disposition index.

The Disposition Index (DI) was greater in MODY3 participants than T2D. We observed that the DI in MODY3 was similar to the DI in controls, because of greater insulin sensitivity in MODY3 that seemed to counterbalance the diminished insulin secretion capability (Fig 2).

### Lipids regulation and suppression indices (Table 2, Fig 1D and 1E)

In the fasting state, MODY3 had lower triglycerides than T2D (Median [IQR] 0.66 nmol/L [0.46–1.15] vs 2.26 [1.65–2.73]; *P* = 0.008). Compared to controls, MODY3 participants tended to have lower triglycerides and showed median fasting NEFA levels twofold higher than controls, but this was not statistically significant. In the post-meal period, it is expected that NEFA are suppressed by insulin in circulation, as illustrated in graphic 1D. MODY3 participants showed a more profound NEFA suppression in early post-meal response as shown by the index ΔNEFA (30–0)/Δinsulin (30–0) (median [IQR] = -39.0 [-78.0; -30.0] *10^4^ (mM/pM)) compared to controls (-3.4 [-4.7; -2.0]; *P* = 0.003) or T2D (-2.0 [-0.61; 6.4] *P* = 0.003). The index ΔNEFA (30–0)/ΔC-peptide (30–0) showed a similar pattern (MODY3 median [IQR] = -0.93 [-1.60; -0.53] mM/nM *vs* controls -0.13 [-0.24; -0.08]; *P* = 0.003 *vs* T2D -0.19 [-0.43; -0.02]; *P* = 0.02). Based on the post-meal excursion graphic, we observed that NEFA in the MODY3 group tended to start at a higher level at fasting, showed a larger drop during the first 2 hours post-meal and an exaggerated rise–between 2 hours to 5 hours post ingestion–which seemed uncharacteristic in the other groups. The overall mealtime AUC-NEFA tended to be higher in MODY3 group compared to the other groups, but this difference was not statistically significant. MODY3 participants had lower AUC-triglycerides compared to T2D (median [IQR] 350.4 [280.5; 484.8] mmol/L *vs* 971.6 [617.3; 1178.9]; *P* = 0.008).

### Ghrelin and incretins (Table 2 and Fig 1F)

We found no statistical difference in ghrelin levels either in fasting or post-meal (AUC-Acylated ghrelin) conditions between any groups. We also found no significant difference in GIP or GLP1, either fasting or post-meal, between MODY3 and control groups. However, T2D had higher total fasting GLP-1 than MODY3 participants.

## Discussion

We found differences in fasting and post-meal glycemic, insulin, and lipid profiles in MODY3 participants compared to healthy subjects and T2D patients. However, in contrast to our hypothesis based on an animal model[18], we did not find differences in ghrelin levels between MODY3 and controls. We also found little difference in incretin levels between MODY3 and other groups.

The post-meal excursion of NEFA showed a more profound suppression in early meal response followed by an exaggerated rise in the late post-prandial period (>210 minutes) in MODY3 compared to the two other groups. In both MODY3 and control groups, triglyceride levels remained lower than in T2D in the postprandial period. The high NEFA–low triglycerides in the fasting state and later post-prandial profile observed in MODY3 is unlikely the consequence of diabetes status since T2D patients actually showed the opposite profile (higher triglycerides–normal/low NEFA). A possible explanation for the observed fasting to postprandial NEFA response in MODY3 is the hypo-insulinemic state resulting in exacerbated intracellular lipolysis in adipose tissues during fasting and late postprandial phase, combined with enhanced adipose tissue insulin sensitivity resulting in more profound inhibitory response of intracellular lipolysis to early postprandial insulin excursion. Some studies have suggested that adipose tissue flexibility may depend on sensitivity to both insulin and catecholamines [27]. This metabolic flexibility of adipose tissues is lost in subjects with pre-diabetes and T2D[28]. It is also possible that other tissues participate in the differential postprandial NEFA response observed in MODY3 participants. Defective *HNF1α* mutations are associated with differential gene expression in tissues other than β-cells. *HNF1α* knockout mice (*HNF1α* -/-) have a different expression of various hepatic genes implicated in lipid metabolism[29] such as overexpression of lipoprotein lipase (LPL) gene transcripts. LPL is localized on the endothelial surface of capillaries of multiple organs such as skeletal muscle, adipose tissues and the heart, where it hydrolyzes circulating triglyceride-rich lipoprotein (VLDL and chylomicrons)[30]. Models of transgenic mice overexpressing LPL have shown a reduction of triglycerides [31–32], in line with what we observed in our MODY3 participants. However NEFA levels have not been reported in this animal model. Moreover, it is important to note that MODY3 have only one defective allele at *HNF1α* and that the phenotype in humans is much less severe than what is seen in homozygote mice (*HNF1α* -/-). The milder phenotype in humans might be explained by compensatory mechanisms in multiple metabolic pathways, including lipid regulation pathways.

HNF1*α* protein in human hepatocytes is believed to bind to several genes involved in lipid metabolism[33]. *HNF1α* defect in humans is associated with increased bile acid synthesis[7] and influences levels of total cholesterol, high density cholesterol and low-density lipoprotein[8], [34]. These effects are likely mediated through regulation of multiple hepatic genes, such as the hepatic lipase promoter gene [35], Niemann-Pick C1-like 1 (*NPC1L1*) and Acyl-CoA: cholesterol acyltransferase-2 (*ACAT-2*) [36]. Also, HNF1*α* enhances transcription of apolipoprotein B and apo(a) and downregulates the transcription of apoCIII and apoA1 which are implicated in metabolism of cholesterol and fatty acids. [37]Therefore, we cannot exclude the involvement of multiple genes implicated in hepatic lipid regulation that might be under HNF1α regulation to explain the divergent lipid profiles.

MODY3 patients are at risk for diabetes complications[19] similarly to T2D but in contrast to patients with GCK mutations (MODY2). Fatty acids induce lipotoxicity in multiple organs and are implicated in the development of diabetes complications[38], including cardiovascular disease[39]. MODY3 patients are known to have lower C-reactive protein and higher HDL circulating levels, features of a healthier cardiometabolic profile. It is currently unknown whether higher NEFA levels observed in fasting state and during late post-prandial period in MODY3 could contribute to the greater risk in cardiovascular complications observed in these patients. Future studies should assess whether NEFA or other lipid particles in MODY3 are predictive of complications, independently of glycemic control. More studies are also needed on metabolic handling of dietary fatty acids[40–42] by adipose and lean tissues in MODY3.

### Acylated ghrelin (fasting and post-meal)

In contrast to our hypothesis derived from diabetic *HNF1α* knockout mice[18], there was no difference between fasting or postprandial acylated ghrelin between MODY3 and control groups. Again, it is important to note that *HNF1α* knockout mice have a more severe phenotype than humans affected by MODY3. Additionally, in contrast to the mice, MODY3 participants in the present study were not all diabetic at the time of the study, because of our inclusion of the two young MODY3 that have not yet reached diabetes status. A study published recently has shown that fasting total ghrelin levels were higher in MODY3 diabetic patients (n = 46) than in the common polygenic forms of diabetes, but lower than in healthy subjects [43]. The difference between the latter and the present study may possibly result from differences in participant selection (all MODY3 participants with overt diabetes) and methods (measurement of total vs. acylated ghrelin levels). It is also possible that the administration of basal insulin the night before by two of the MODY3 participants might have “normalized” their fasting profile, including ghrelin. Studies that reported acylated ghrelin levels in T2D subjects showed lower[16] or higher levels [15–44] compared to normoglycemic individuals. Therefore, whether acylated ghrelin levels are abnormal in T2D is controversial.

### Total GIP and GLP-1

We found no difference in fasting or postprandial total GIP and GLP-1 levels between MODY3 and controls. Only one previous study has also reported incretin levels in MODY3 and found essentially no difference[45]. We found no statistical differences in GIP and GLP-1 postprandial levels in T2D compared to controls, in line with previous reports and recent meta-analyses[46–48]. However in our study, T2D had higher fasting incretin levels than controls. This increase in fasting incretin levels might be a compensatory mechanism to overcome the incretin resistance of the β-cell that is a possible finding in the early stages of T2D[49].

### Strengths and limitations

**Fig 3:**
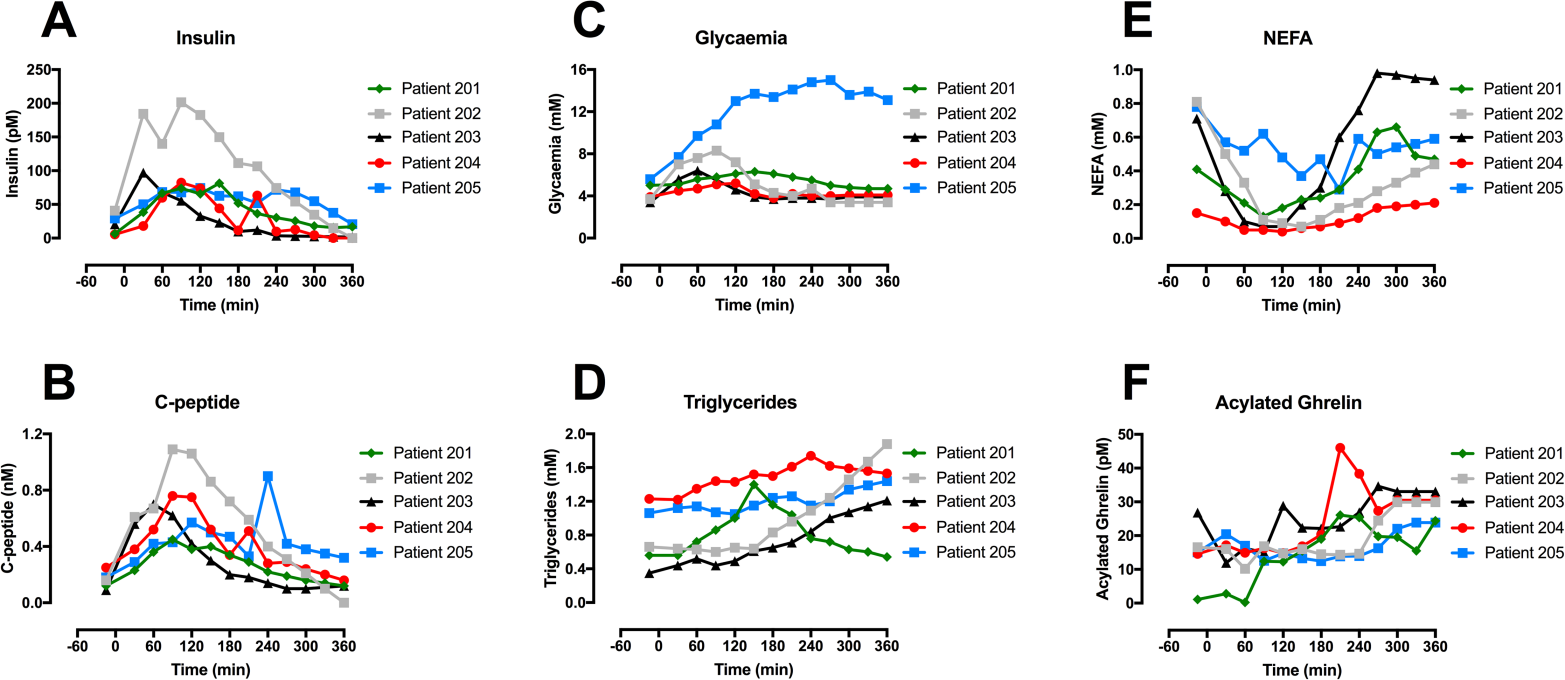
Post-meal excursion curves of biomarkers in all HNF1α carriers, including two non-diabetic HNF1α children (#203 and #204). (A) Insulin (B) C-peptide (C) Glycemia (D) NEFA (E) Triglycerides (F) Acylated Ghrelin.

Among our strengths, we used a standardised liquid meal to simulate the post-meal hormonal excursion in response to a mix meal. Therefore, the postprandial state in the present study is more physiological than could have been obtained using oral glucose challenge. Another strength is the repeated measures of multiple hormones throughout the post-meal period, up to 6h after ingestion of the meal. The main limitation of our study, however, is its small sample size, limiting our power to detect some probable differences in postprandial hormonal responses between groups. It is therefore impossible to rule out small (i.e. less than 30%) differences in insulin, C-peptide, GLP-1, GIP and ghrelin excursion between MODY3 and the two other groups. Our small sample size also precluded us from assessing the influence of age and sex on our results using multivariate regression analyses; given that our groups differed in age and sex distributions, we cannot exclude that some of our results may be confounded by these characteristics. Nevertheless, although two MODY3 were younger (5 and 10 years old), these participants were not outliers with regards to the observed metabolic changes we found in this group (Fig 3). Despite our small sample size, we detected the expected defect in insulin secretion, and clearly different patterns in lipid profiles. Another limitation of our study is the use of metformin in participants with T2D that may have affected some of the metabolic outcomes, including level of incretin hormones and ghrelin. Two of the MODY3 patients were instructed to take their usual long-acting insulin dose the night before metabolic testing to prevent marked hyperglycemia and we acknowledge that this might have affected some of the fasting profiles; however, this should not have influenced the estimation of insulin secretion as none of the MODY3 patients injected short acting insulin the morning of the meal test.

In conclusion, MODY3 participants showed a greater early post-prandial NEFA suppression—despite reduced insulin secretion–followed by higher NEFA excursion in late post-prandial state. MODY3 participants also maintained very low post-prandial triglycerides levels. Based on previous literature and on the observation that our MODY3 participants showed greater insulin sensitivity, we hypothesized that this post-prandial pattern of lipid regulation might be explained by better adipose tissue metabolic flexibility. It is also possible that differential expressions of lipid metabolism genes that are under control of HNF1*a* transcription factors–especially in the liver—are responsible for the observed post-prandial differences. Future mechanistic studies will be necessary to reveal the causes of differential post-prandial lipid regulation in MODY3 patients.
